# Efficient Cas9-based genome editing of *Rhodobacter sphaeroides* for metabolic engineering

**DOI:** 10.1186/s12934-019-1255-1

**Published:** 2019-11-25

**Authors:** Ioannis Mougiakos, Enrico Orsi, Mohammad Rifqi Ghiffary, Wilbert Post, Alberto de Maria, Belén Adiego-Perez, Servé W. M. Kengen, Ruud A. Weusthuis, John van der Oost

**Affiliations:** 1 Laboratory of Microbiology, Wageningen University, Stippeneng 4, 6708 WE Wageningen, The Netherlands; 2 Bioprocess Engineering, Wageningen University, Droevendaalsesteeg 1, 6708 PB Wageningen, The Netherlands; 3 Present Address: Department of Chemical and Biomolecular Engineering, Korea Advanced Institute of Science and Technology, Daejeon, South Korea; 4 Present Address: Systems and Synthetic Metabolism Group, Max Planck Institute of Molecular Plant Physiology, Am Mühlenberg 1, 14476 Potsdam, Germany

**Keywords:** *Rhodobacter sphaeroides*, Cas9, Genome editing, PHB

## Abstract

**Background:**

*Rhodobacter sphaeroides* is a metabolically versatile bacterium that serves as a model for analysis of photosynthesis, hydrogen production and terpene biosynthesis. The elimination of by-products formation, such as poly-β-hydroxybutyrate (PHB), has been an important metabolic engineering target for *R. sphaeroide*s. However, the lack of efficient markerless genome editing tools for *R. sphaeroides* is a bottleneck for fundamental studies and biotechnological exploitation. The Cas9 RNA-guided DNA-endonuclease from the type II CRISPR-Cas system of *Streptococcus pyogenes* (SpCas9) has been extensively employed for the development of genome engineering tools for prokaryotes and eukaryotes, but not for *R. sphaeroides*.

**Results:**

Here we describe the development of a highly efficient SpCas9-based genomic DNA targeting system for *R. sphaeroides*, which we combine with plasmid-borne homologous recombination (HR) templates developing a Cas9-based markerless and time-effective genome editing tool. We further employ the tool for knocking-out the uracil phosphoribosyltransferase (*upp*) *gene* from the genome of *R. sphaeroides,* as well as knocking it back in while altering its start codon. These proof-of-principle processes resulted in editing efficiencies of up to 100% for the knock-out yet less than 15% for the knock-in. We subsequently employed the developed genome editing tool for the consecutive deletion of the two predicted acetoacetyl-CoA reductase genes *phaB* and *phbB* in the genome of *R. sphaeroides*. The culturing of the constructed knock-out strains under PHB producing conditions showed that PHB biosynthesis is supported only by PhaB, while the growth of the *R. sphaeroides* Δ*phbB* strains under the same conditions is only slightly affected.

**Conclusions:**

In this study, we combine the SpCas9 targeting activity with the native homologous recombination (HR) mechanism of *R. sphaeroides* for the development of a genome editing tool. We further employ the developed tool for the elucidation of the PHB production pathway of *R. sphaeroides.* We anticipate that the presented work will accelerate molecular research with *R. sphaeroides.*

## Introduction

The purple non-sulphur bacterium *Rhodobacter sphaeroides* is a microorganism with an extremely adaptable metabolism [1]. It is a facultative phototroph that can grow on many different carbon substrates and can respire aerobically and anaerobically with different electron acceptors [2, 3]. The metabolic versatility of *Rhodobacter sphaeroides* has raised curiosity for white biotechnological applications. In particular, this microorganism has been largely studied for photoheterotrophic hydrogen production and chemoheterotrophic terpene biosynthesis. In both processes, approaches for improving the metabolic performances of the microorganism were investigated [4–8]. Reducing formation of by-products like the polymer poly-β-hydroxybutyrate (PHB) holds potential for eliminating competition for carbon and reducing power, as already proven to work for H_2_ biosynthesis [5, 9]. Nonetheless, a lot of work is still required to further increase our knowledge on *R. sphaeroides* metabolism and exploit its biotechnological potential.

The high-throughput exploration of *R. sphaeroides* metabolism requires the development of highly effective and time-efficient genome engineering tools [4]. Currently, the introduction of genomic modifications *in R. sphaeroides* is based on suicide-plasmid driven homologous recombination (HR) systems [10, 11]. These systems depend on the incorporation of the non-replicating vector into the *R. sphaeroides* genome via an initial single crossover (SCO) event and consequent excision of the vector via a second SCO event upon induction of counter-selection pressure i.e. employing a levansucrase gene (*sacB*) carrying vector [11–17]. The second SCO can either lead to the reconstitution of a wild type genomic background or to the desired genetic modification [11]. The lack of a strict counter-selection method targeting specifically the genomic region of interest usually leads to high rates of wild-type revertants, especially for essential genes. Therefore, screening of mutants can be time consuming and is frequently unsuccessful.

A wide range of CRISPR-Cas (Clustered Regularly Interspaced Short Palindromic Repeats-CRISPR associated proteins) bacterial and archaeal adaptive immune systems have been repurposed as tools for eukaryotic, prokaryotic and archaeal genome engineering [18–26]. Most of these tools exploit the RNA-guided DNA endonuclease from the type II CRISPR-Cas system of *Streptococcus pyogenes,* denoted as SpCas9. As any Cas9 orthologue, SpCas9 can be easily programmed to precisely introduce double stranded DNA breaks (DSB) to a selected DNA sequence, denoted as protospacer. A single, customizable guide RNA (sgRNA) molecule directs Cas9 to the protospacer via complementarity between its exchangeable 5′-end sequence, denoted as spacer, and the protospacer sequence. The only additional requirement for Cas9-based targeting is the presence of a specific, 2–8 nt long motif right after the 3′-end of the selected protospacer, denoted as protospacer adjacent motif (PAM) [27]. All in all, the simplicity in the design and construction of a Cas9-based DNA targeting system has made it popular as the basis for numerous genome manipulation applications.

A plethora of Cas9-based tools have been developed the last 5 years for prokaryotes [24, 28–33]. Cas9 has been extensively used as an efficient HR induction and counter selection tool, when combined with plasmid-based homologous recombination (HR) or recombineering; alternative DSB repairing mechanisms (like the template independent non-homologous end joining, NHEJ) are lacking in most prokaryotes [34]. Therefore, Cas9-based DSBs after HR are lethal if introduced to the wild type genomes: unmodified cells will be eliminated from the treated population, allowing survival only for the recombined ones [34].

In this study, we develop a highly efficient Cas9-based targeting system for *R. sphaeroides*. We further combine it with HR templates, developing an HR-Cas9 counter selection tool. We then employ the tool for the proof-of-principle efficient generation of uracil-phosphoribosyltransferase (*upp*) gene knock-out and knock-in strains, the latter combined with single nucleotide substitution. The developed process simplifies and accelerates *R. sphaeroides* genome editing, as it requires only 3 days from conjugation to screening of clean mutants. Additionally, we use the developed tool for the construction of acetoacetyl-CoA reductase deletion mutant strains, elucidating the dominant metabolic pathway of *R. sphaeroides* towards PHB production.

## Materials and methods

### Bacterial strains, media and growth conditions

The strains used in this study are listed in Table 1. *E. coli* DH5a was used for cloning and routine amplification. *E. coli* S17-1 was used as vector donor for *R. sphaeroides* in diparental conjugation.

**Table 1:** List of strains used in this study

Strain	Description	Source of reference
*E. coli*		
DH5α	fhuA2 Δ(argF-lacZ)U169 phoA glnV44 Φ80 Δ(lacZ)M15 gyrA96 recA1 relA1 endA1 thi-1 hsdR17Strain for assembly and plasmid amplification	Laboratory stock
S17-1	Host strain for transconjugation, *thi pro recA hsdR* [RP4-2Tc::Mu-Km::Tn7] Tp^r^ Sm^r^	Laboratory stock
*R. sphaeroides*		
265-9c	Wild type	Derivative of ATCC 35053
Δ*upp*	265-9c Δ*upp*	This study
*upp*KI	265-9c KI(ATG->GTG)*upp*	This study
Δ*phaB*	265-9c Δ*phaB*	This study
Δ*phbB*	265-9c Δ*phbB*	This study
Δ*phaB*_Δ*phbB*	265-9c Δ*phaB*-Δ*phbB*	This study

Culturing of *R. sphaeroides* was performed in RÄ minimal or LB (Luria–Bertani) medium at 250 rpm, 30 °C. Growth on solid medium was performed on RÄ supplemented with 15% w/v agar. Culturing of *E. coli* strains was performed in LB medium at 250 rpm, 37 °C. Growth on solid medium was performed on LB supplemented with 15% w/v agar. When required, kanamycin (50 µg/mL) was added for all the mentioned growing conditions.

### Plasmids construction

The plasmids constructed and the primers used for cloning and sequencing are listed in Additional file 1: Tables S1 and S2, respectively. All primers for PCR were prepared by Integrated DNA Technologies (IDT) and all the PCR amplifications were performed using Q5 High-Fidelity DNA polymerase Master Mix 2× from New England Biolabs (NEB) according to the manufacturer’s protocol. Due to the high GC content of *R. sphaeroides* genome, all PCR reactions using its genomic DNA as template were supplemented with DMSO 3% v/v. PCR fragment assemblies were performed employing the HiFi Assembly kit (NEB). Prior to plasmids assemblies, all PCR products were purified via gel extraction using the Zymoclean Gel DNA Recovery Kit (Zymo Research). Plasmid extractions were performed using the GeneJET Plasmid Miniprep kit (Thermo Fisher Scientific) and *R. sphaeroides* genome extractions were performed using the GeneJET Genomic DNA Purification Kit (Thermo Fisher Scientific).

### Generation of the non-targeting Cas9 plasmid

A synthetic construct containing the sgRNA construct (promoter BBa_J95023, sgRNA scaffold and BBa_J95029 terminator) (Additional file 1: Table S3) was ordered as gBlock from Integrated DNA Technologies (Leuven, Belgium). After restriction digestion with SmaI and SalI (Thermo Fisher Scientific), the sgRNA construct was ligated into the pUC19 vector and transformed into *E. coli* TOP10 cells for storage and amplification. Subsequently, the pUC19 containing the sgRNA construct and the pBBR1MCS2 *E. coli*-*R. sphaeroides* shuttle vector were digested with the same restriction enzymes. After digestion, the sgRNA construct and the pBBR1MCS2 backbone were ligated with T4 ligase (Thermo Fisher Scientific) yielding the pBBR1MCS2-sgRNA. The *cas9* gene was codon-harmonized according to *R. sphaeroides* codon-usage using the Galaxy/Codon harmonizer on-line tool [35]. The codon-harmonized *cas9* was synthetically constructed by Baseclear (the Netherlands) (Additional file 1: Table S4) with a 6×His-tag fused at its C-terminus, and delivered inserted in a pUC57 vector backbone. The *cas9* gene was PCR amplified using the pUC57hSpCas9 vector as template and primers BG10937/BG10938. The pBBR-sgRNA vector was linearized using primers BG10939/BG10941. The construction of the non-targeting plasmid pBBR_Cas9_NT vector (Additional file 1: Figure S1) was done via HiFi assembly of the two aforementioned PCR amplicons and the *cas9* amplicon was inserted downstream of the lac promoter site of the pBBR-sgRNA. The lack of *lacI* repressor gene or its homolog in *R. sphaeroides* genome allowed constitutive transcription of the harmonized *cas9* gene.

### Generation of the *upp* targeting plasmids

The spacers introduced in the sgRNA construct of the pBBR_Cas9 vectors are listed in the Additional file 1: Table S5. The spacers were selected employing the chopchop web tool (https://chopchop.cbu.uib.no/). The spacer- selection parameters were set to avoid self-complementarity of the sgRNA, have efficiency more than 50 and no predicted off-targets. Using the plasmid pBBR_Cas9_NT as template, it was possible to substitute the non-targeting spacer contained in the sgRNA construct with three different targeting spacers for the gene uracil phosphoribosyltransferase *upp* (sp1–sp3). The pBBR_Cas9_NT was used as template for PCR amplification of the backbone, the start codon and part of the *cas9* gene, employing primers BG11415/BG11416. The remaining *cas9* sequence and the promoter of the sgRNA construct were PCR amplified employing primer BG11412 in combination with primers BG11486, BG11487 and BG11411, which also contained the spacer 1, 2 and 3 sequences, respectively as overhangs. The remaining part of the backbone and the constant part of the sgRNA construct were PCR amplified employing primer BG11413 in combination with primers BG11488, BG11489 and BG11414 which also contained the spacer 1, 2 and 3 sequences, respectively as overhangs. 3 fragments assemblies were performed and the reaction mixtures were transformed in DH5α *E. coli* competent cells (NEB) via heat shock, for storage and plasmid amplification. Upon sequence verification, the pBBR_Cas9_NT plasmid was transformed to *E. coli* S17-1 cells.

### Generation of vectors for gene knock-outs (KO) and gene knock-in (KI)

Homologous recombination (HR) followed by Cas9-based counter selection was used for the KO of the *upp* gene, as well as the KI of the *upp* gene back to its original place in the *R. sphaeroides* genome combined with single nucleotide substitution.

### Plasmids for *upp* KO

We started by constructing the HR control, non-targeting plasmids pBBR_Cas9_Δupp500HR_NT and pBBR_Cas9_Δupp1000HR_NT. The pBBR_Cas9_NT backbone was PCR linearized into two fragments using the primers sets BG11886/BG11182 and BG11887/BG11888. The 500 bp or 1 kb long upstream and downstream flanking sites of the *upp* gene were PCR amplified from the *R. sphaeroides* genome employing the primer sets BG11866/BG11867 and BG11869/BG11871 for the 500 bp sizes and the primer sets BG11866/BG11868 and BG11870/BG11871 for the 1 kb sizes. Hifi assembly and transformation to *E. coli* DH5a cells was followed by sequence verification of the obtained pBBR_Cas9_Δupp500HR_NT and pBBR_Cas9_Δupp1000HR_NT plasmids. The sequence verified plasmids was transformed to *E. coli* S17-1 cells.

We proceeded with the construction of the HR-editing, *upp* targeting plasmids pBBR_Cas9_Δupp500HR_sp2 and pBBR_Cas9_Δupp1000HR_sp2. The previously mentioned couples of primer sets were used for the generation of the 500 bp and 1 kb HR flanking sites. The pBBR_Cas9_NT vector was employed as template for the PCR amplification of the conserved backbone region with the BG11887/BG11888 primer set. In addition, the spacer sp2 was included in the corresponding position employing the primer sets BG11182/BG11487 and BG11489/BG11886 for the amplification of the *cas9* gene and the sgRNA module, respectively. Hifi assembly and transformation to *E. coli* DH5a cells was followed by sequence verification of the obtained pBBR_Cas9_Δupp500HR_sp2 and pBBR_Cas9_Δupp1000HR_sp2 plasmids. The sequence verified plasmids were transformed to *E. coli* S17-1 cells.

### Plasmids for *upp* KI

We proceeded with the construction of the *upp* knock-in (KI) control pBBR_Cas9_KIupp1000HR_NT plasmid and the *upp* KI plasmids pBBR_Cas9_KIupp1000HR_sp4 and pBBR_Cas9_KIupp1000HR_sp5. The pBBR_Cas9_NT plasmid was used as template for the amplification of the fragments containing the backbone (primer set BG11887/BG11888), the *cas9* gene and the targeting sgRNAs. The *cas9*- and the sgRNA-containing fragments were generated via PCR amplification with primer sets BG11182/BG12907 and BG11186/BG12908 for spacer sp4 and primer sets BG11182/BG12909 and BG11186/BG12910 for spacer 5. Additionally, the HR template for KI of the *upp* gene back to its native position were comprised of the *upp* gene sequence flanked by the 1000 bp genomic regions upstream and downstream of the *upp* gene. The genomic amplification for the 1000-bp long flanking sites was performed using the primer pairs BG12347/BG11868 and BG11870/BG12348. The primers BG12347 and BG12348 introduce a single point mutation in the *upp* start codon (ATG to GTG). The construction of the two editing plasmids, namely pBBR_Cas9_KIupp1000HR_sp4 and pBBR_Cas9_KIupp1000HR_sp5, was performed via 5-fragment assemblies. The assembly mixtures were then transformed to *E. coli* DH5α cells. Upon sequence verification, the plasmids were transformed to *E. coli* S17.

### Plasmids for *phaB* KO

We employed the previously described cloning strategy for generating three *phaB* KO plasmids (pBBR_Cas9_ΔphaBHR_sp1, pBBR_Cas9_ΔphaBHR_sp2 and pBBR_Cas9_ΔphaBHR_sp3). The pBBR_Cas9_NT plasmid was used as template for the amplification of the fragments containing the backbone (primer set P302/P303), the *cas9* and the targeting sgRNAs. Each plasmid carried a different spacer designed to target a protospacer within the *phaB* sequence. For spacer sp1, the *cas9*- and the sgRNA-containing fragments were generated via PCR amplification with primer sets P301/P383 and P304/P384, respectively. For spacer sp2 the *cas9*- and the sgRNA-containing fragments were generated via PCR amplification with primer sets P301/P385 and P304/P386, respectively. For spacer sp3 the *cas9*- and the sgRNA-containing fragments were generated via PCR amplification with primer sets P301/P387 and P304/P388, respectively. Each of the three *phaB* editing plasmids contained the same HR template, consisted of the genomic regions 1 kb upstream of the *phaB* start codon and 1 kb downstream of the *phaB* stop codon. The regions were generated via PCR amplification with primer sets P379/P380 and P381/P382, respectively. The construction of the three editing plasmids was performed via 5-fragment assemblies. The assembly mixtures were then transformed to *E. coli* S17 cells and the constructed plasmids were sequence verified.

We employed the previously described cloning strategy for generating three *phbB* KO plasmids (pBBR_Cas9_ΔphbBHR_sp1 and pBBR_Cas9_ΔphbBHR_sp2). The pBBR_Cas9_NT plasmid was used as template for the amplification of the fragments containing the backbone (primer set P302/P303), the *cas9* and the targeting sgRNAs. Each plasmid carried a different spacer designed to target a protospacer within the removed *phbB* sequence. For spacer sp1, the *cas9*- and the sgRNA-containing fragments were generated via PCR amplification with primer sets P301/P418 and P304/P417, respectively. For spacer sp2 the *SpCas9*- and the sgRNA-containing fragments were generated via PCR amplification with primer sets P301/P420 and P304/P419, respectively. Each of the two *phbB* editing plasmids contained the same HR template, consisted of a sequence of four stop codons (TAG) and an EcoRI restriction site, flanked on the one side by the genomic region 500 bp upstream and 546 bp downstream the *phbB* start codon and on the other side by the genomic region 145 bp upstream and 850 bp downstream the *phbB* stop codon. The regions were generated via PCR amplification with primer sets P415/P416 and P413/P414, respectively, using the *R. sphaeroides* genome as template. Moreover, each plasmid carried a different spacer designed to target a protospacer within the 59 nt long substituted *phbB* region. The construction of the two editing plasmids was performed via 5-fragment assemblies. The assembly mixtures were then transformed to *E. coli* S17 cells and the constructed plasmids were sequence verified.

### Diparental conjugation and plasmid curation

*Rhodbacter sphaeroides* was inoculated from glycerol stock in 10 mL RÄ medium and incubated for 2 days at 30 °C and 200 rpm prior to conjugation. After 48 h, 50 μL of culture were transferred to fresh 10 mL RÄ medium, incubated for 24 h under the same conditions, and harvested when the OD at 600 nm was approximately 3. *E. coli* S17-1 harbouring the desired plasmid was grown overnight in LB supplemented with kanamycin at 37 °C and 200 RPM. Then, the overnight culture was transferred to fresh 2xYT media with antibiotic and grown for 2 h until the OD at 600 nm was approximately 1. The cell suspension was harvested and washed twice with 1 mL of RÄ medium, mixed with *R. sphaeroides* culture at a 1:1 ratio (1 mL each) and centrifuged for 1 min (maximum speed). The pellet was concentrated by suspension in 100 μL of RÄ medium, which was transferred to a sterile 0.22 µm 47 mm diameter nitrocellulose filter disc (Sigma-Aldrich) on a PY agar plate and incubated for 6 h at 28 °C. The conjugation mixture was harvested and re-suspended in 2 mL of RÄ medium. 100 μL of diluted culture was spread on RÄ agar plates containing kanamycin and incubated at 30 °C for 3 days until colonies appeared. Purification of *R. sphaeroides* conjugants from *E. coli* S17-1 donors was performed by subsequent transfer of *R. sphaeroides* colonies to LB agar medium supplemented with kanamycin until no *E. coli* colonies appeared. Finally, a colony PCR was performed using primer set P3/P4 to verify that *E. coli* was not present in the culture.

Plasmid curing from positive *R. sphaeroides* mutants was carried out by cultivating the mutants in 5 mL of RÄ medium without antibiotic for 24 h, twice. The cells from the second cultivation were then diluted and spread on RÄ agar plates. The individual colonies were streaked onto LB agar with and without kanamycin. Those cells that grew on LB without antibiotic but were unable to grow in the presence of antibiotic were considered to have lost the plasmid. Final verification of plasmid curing was conducted by performing PCR with primers BG10937/BG10938, which are specific for the Cas9 gene.

### RNA extraction and reverse transcriptase PCR (RT-PCR)

To check expression of the *cas9* gene under the lac promoter, total RNA of *R. sphaeroides* conjugated with the pBBR_Cas9_NT plasmid was extracted employing the RNAeasy mini kit (Qiagen, Germany) according to the manufacturer’s protocol and treated with DNAse I (NEB) to remove genomic DNA contamination in the sample. The SuperScript III Reverse Transcriptase kit (Invitrogen) was used for RT-PCR. When the first strand of cDNA was synthesized, the primers BG11112 and BG11115 were used to verify transcription activity of Cas9 gene using standard PCR.

### 5-FU screening

For the 5-fluorouracil (5-FU) screening process we prepared RÄ agar plates supplemented with 5-FU up to 100 μg/mL final concentration, using a 50 mg/mL stock solution prepared in dimethyl sulfoxide (DMSO). Colonies obtained from conjugation were picked and streaked on RÄ_5-FU plates with kanamycin. Survival colonies were isolated and their *upp* locus was then PCR amplified and sequenced.

### Analytics

#### Carbon and nitrogen concentrations

To determine active biomass levels and C/N ratios of the biomass, analysis was done using a TOC-L analyzer (Shimadzu). Biomass samples were diluted 3–5 times in a total volume of 15 mL using MilliQ and a stirring bar was added. The TOC-L analysed both non-purgeable organic carbon (NPOC) as well as total nitrogen (TN) content. The TN content was used to calculate the active biomass concentration using the elemental composition of CH_1.99_O_0.5_N_0.19_ of *R. sphaeroides* described previously [36]. The C/N ratio was calculated by dividing molar concentrations resulting from NPOC and TN measurements.

#### Polyhydroxybutyrate (PHB) concentrations

Volumes ranging from 2 to 5 mL of biomass were spun down and resuspended in MilliQ water. This was spun down and pellets were dried in an oven at 100 °C. The dried biomass was resuspended in 1 mL of concentrated H_2_SO_4_ and heated to 90 °C for 40 min. The samples were diluted by addition of 9 mL of 0.09 mM H_2_SO_4_. Analogous to the organic acid analysis, samples were mixed 1:1 with propionic acid. Separation of compounds was done using the same flow and temperature as the organic acids. For quantification, standards with known concentrations of commercially available PHB (Sigma-Aldrich) were treated the same as the samples.

## Results and discussion

### SpCas9 targeting in *R. sphaeroides*

**Fig. 1:**
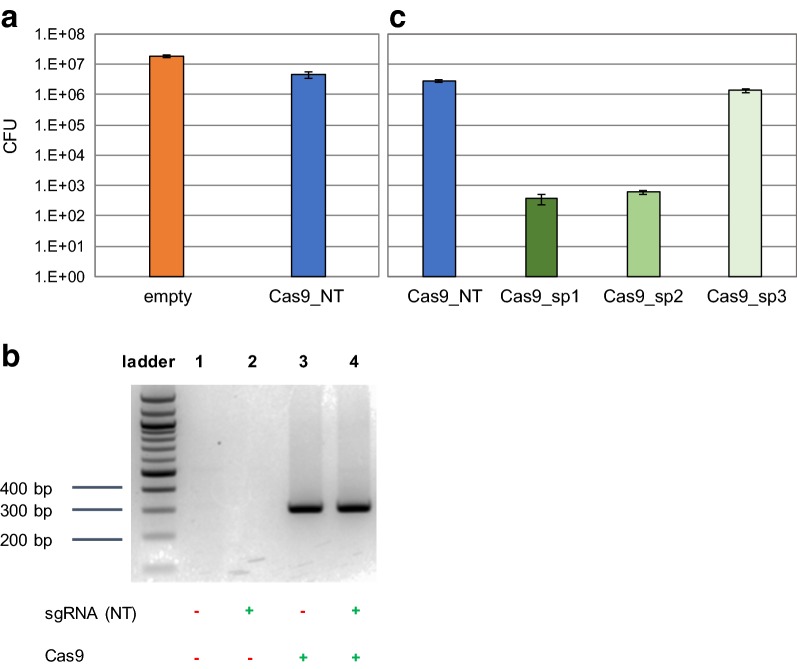
Conjugation of *upp* targeting Cas9 plasmids and Cas9 transcription. **a** Colony forming units (CFUs) obtained after conjugation of pBBR_Cas9_NT plasmid in *R. sphaeroides*. The efficiency was compared to an empty pBBR1MCS2 plasmid. The average values of 3 replicates are shown and the error bars represent S.D. **b** Reverse Transcriptase PCR (RT-PCR) assessing the transcription of the *cas9* gene under the constitutive P_*lac*_ promoter in *R. sphaeoroides*. *R. sphaeroides* cells were conjugated with pBBR1MCS2 harbouring: no additional features (well 1), non-targeting sgRNA (pBBR_NT, well 2), harmonized Cas9 (pBBR_Cas9, well 3) and harmonized Cas9 plus NT-sgRNA (pBBR_Cas9_NT, well 4). The expected size of the *cas9* cDNA amplicon is 307 bp. The primers set used was BG11112/BG11115. **c** CFU obtained after conjugation of the pBBR_Cas9 plasmids harbouring different *upp* targeting spacers (pBBR_Cas9_sp1–sp3); the plasmid with the non-targeting spacer (pBBR_Cas9_NT) was used as control. The average values of 3 replicates are shown and the error bars represent S.D.

The first aim of this study was to assess potential toxic effects of SpCas9 expression in *R. sphaeroides* cells, as previously reported for other microbial species [37–39]. For this purpose, we constructed the pBBR_Cas9_NT control vector by cloning the *cas9* gene, codon optimized for *R. sphaeroides*, and a sgRNA expressing module with a non-targeting (NT) spacer in the *E. coli*-*Rhodobacter* shuttle vector pBBR1MCS2. The expression of the *cas9* gene was under control of the *P*_*lac*_ promoter that due to the absence of the *lacI* repressor gene in the *R. sphaeroides* genome, has constitutive transcription activity. Moreover the sgRNA expressing module was under the control of the synthetic constitutive *P*_*BBa_J95023*_ promoter [40] and the NT spacer sequence was designed in such way that any candidate protospacer within the genome of *R. sphaeroides* would contain at least 6 mismatches in the non-seed region (the 12 PAM-distal nucleotides) and at least 2 extra miss-matches in its seed region (the 8 PAM-proximal nucleotides). The pBBR_Cas9_NT vector in parallel with the pBBR1MCS2 control vector were conjugated in *R. sphaeroides* cells (Additional file 1: Figure S2). Even though the conjugation efficiency for the pBBR_Cas9_NT vector was reduced compared to the control pBBR1MC2 vector (Fig. 1a), it remained high and the size of the pBBR_Cas9_NT containing colonies was comparable to the size of the pBBR1MC2 containing colonies. We then randomly selected 5 of the colonies conjugated with the pBBR_Cas9_NT vector for culturing and plasmid isolation. The sequence integrity of the *cas9* gene, the sgRNA expressing locus and the corresponding promoters was verified for all the 5 isolated plasmids, leading us to the conclusion that the drop in the conjugation efficiency of the pBBR_Cas9_NT can most likely be attributed to the almost double size of the vector (9566 bp) compared to the control vector (5144 bp) and, at least under the tested conditions, not to toxicity of SpCas9. Moreover, we confirmed the transcription of the *cas9* gene in *R. sphaeroides* by RT-PCR (Fig. 1b).

We further developed a SpCas9-based system for efficient introduction of lethal double stranded DNA breaks (DSBs) in *R. sphaeroides*, genome, selecting the *upp* (uracil phosphoribosyltransferase) gene as the proof of principal target. Knock-out mutations of the *upp* gene in other bacteria resulted in mutant strains resistant to 5-fluorouracil [41, 42]. For *R. sphaeroides* we determined that 5-Fluorouracil is toxic at concentrations as low as 1 μg/mL in RÄ medium and its use could facilitate future screening steps. The pBBR_Cas9_NT vector was employed for the construction of three targeting plasmids (pBBR_Cas9_sp1-3) each containing a unique spacer that corresponds to a different target sequence (protospacer) within the *upp* gene. if the constructed SpCas9 system is efficiently expressed in *R. sphaeroides*, the number of obtained colonies upon conjugation with the targeting plasmids is going to be substantially lower compared to the number of obtained colonies upon conjugation with the control plasmid.

The pBBR_Cas9 series of plasmids was conjugated in *R. sphaeroides* (Fig. 1c). The conjugation efficiency for the pBBR_Cas9_sp1 and pBBR_Cas9_sp2 constructs dropped more than 3 orders of magnitude compared to the pBBR_Cas9_NT control, while for the pBBR_Cas9_sp3 the conjugation efficiency dropped only by 50% (Fig. 1c). This result shows that the constructed SpCas9 targeting system is highly active in *R. sphaeroides*. To further study the observed fluctuation of targeting efficiency of different spacers, we randomly selected 5 surviving colonies conjugated with the pBBR_Cas9_sp3 plasmid, isolated the plasmids. The plasmid sequencing results did not reveal any indel mutations or single nucleotide substitutions in the *cas9* gene and the sgRNA module that could lead to the deactivation of the SpCas9 targeting system. Previous studies have shown that CRISPR-Cas based chromosomal targeting can induce genomic island excision events, resulting to the removal of the chromosomal target in the surviving population [43, 44]. We PCR amplified the *upp* genomic regions from the previously selected 5 surviving colonies conjugated with the pBBR_Cas9_sp3 plasmid; all the amplicons showed the expected wild-type size (Additional file 1: Figure S3) and were also sequence verified. This result excluded a genomic island excision event. It was previously reported that the targeting efficiency of a SpCas9 system relies heavily on the selected spacer and can substantially differentiate amongst different targets, even within the same gene [45]. Hence, the most possible explanation for the low targeting efficiency of the SpCas9-based system employing spacer 3 is the comparatively higher efficiency of the native RecA-based repair mechanism that employs untargeted chromosomal copies as repairing templates [45].

### Homologous recombination-SpCas9 counter selection for gene deletions

There are multiple studies on bacterial genome editing employing (i) homologous recombination (HR) via plasmid-borne templates that carry the desired modifications, and (ii) Cas9-induced DSBs for the induction of the cellular HR machinery and as a counter-selection system for the unedited cells [24, 34]. Previous studies reported HR activity in *R. sphaeroides* [11] and here we developed a SpCas9 targeting system. Hence, we set out to develop a HR-SpCas9 counter selection system for efficient genome editing in *R. sphaeroides*.

As a proof of principle, we programmed the designed HR-SpCas9 system to knock out the *upp gene* of *R. sphaeroides*, excluding the start codon and the last 12 nucleotides of the gene in order to avoid potential polar effects for neighboring genes of the operon. Two editing plasmids were constructed and tested, both containing the previously described spacer 2 for efficient *upp* targeting and HR templates consisted of either 500 bp (pBBR_Cas9_Δupp500HR_sp2) or 1 kb (pBBR_Cas9_Δupp1000HR_sp2) upstream and downstream genomic regions flanking the selected for deletion *upp* fragment. Two control plasmids (pBBR_Cas9_Δupp500HR_NT and pBBR_Cas9_Δupp1000HR_NT), containing the same HR templates as the editing plasmids but a non-targeting spacer, were also taken along for assessing the contribution of SpCas9 to the efficiency of the tool.

**Fig. 2:**
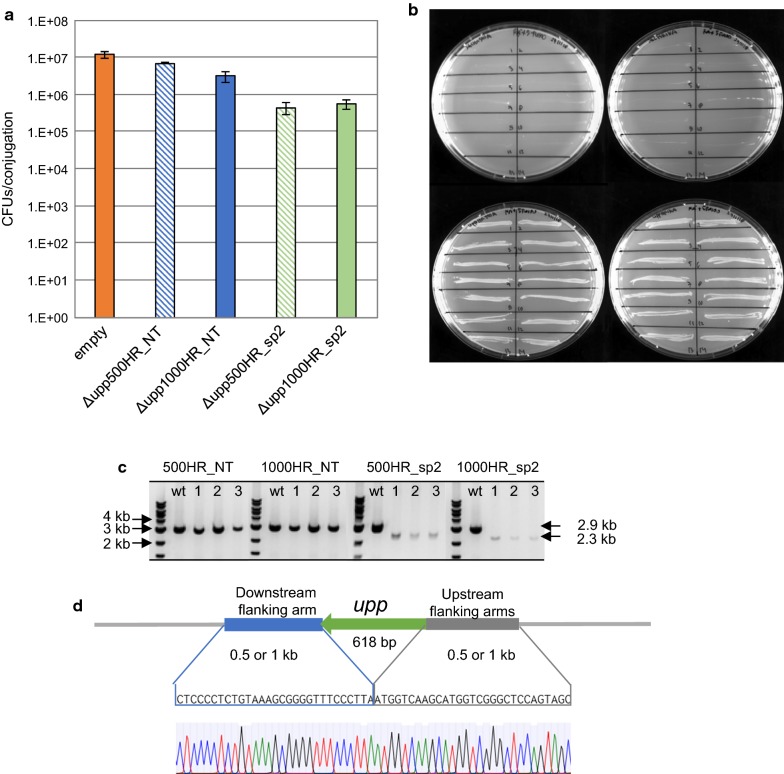
Deletion of *upp* gene from *Rhodobacter sphaeroides* genome. **a** Number of colonies obtained on RÄ agar plates plated with 10^−3^ dilutions of *R. sphaeroides* conjugation mixtures with the homologous recombination (HR) control vectors pBBR_Cas9_Δupp500HR_NT and pBBR_Cas9_Δupp1000HR_NT, and HR editing vectors pBBR_Cas9_Δupp500HR_sp2 and pBBR_Cas9_Δupp1000HR_sp2. The error bars represent standard deviations from three replicate experiments. **b** Restreaks of randomly selected colonies from the above mentioned conjugations on RÄ agar plates supplemented with 5-FU. Only Δ*upp* mutants can grow on 5-FU plates. **c** Genome specific colony PCR amplification of the *upp* locus in cells conjugated with the pBBR_Cas9_Δupp500HR_NT, pBBR_Cas9_Δupp1000HR_NT, pBBR_Cas9_Δupp500HR_sp2 and pBBR_Cas9_Δupp1000HR_sp2 vectors. Amplification yields a 2992 bp product for the wild type *upp* gene and a 2374 bp product for the deleted *upp* gene. **d** Sequence verification of the desired *upp* deletion by Sanger sequencing

Upon conjugation of the above described constructs in *R. sphaeroides*, a drop was observed 1 order of magnitude in the number of surviving colonies upon conjugation with the editing constructs compared to the non-targeting control construct (Fig. 2a). 14 colonies were randomly picked from each plate (the experiment was performed in triplicates, so 42 colonies were selected per conjugated construct) and streaked on RÄ agar plates supplemented with 5-FU. All selected colonies from the conjugation plates with editing constructs -regardless the size of the employed HR template- grew on the 5-FU selection plates (Fig. 2b). We genotyped all the selected colonies through colony PCR and sequencing, and we confirmed the desired clean deletions (Fig. 2c, d). Meanwhile none of the selected colonies from the conjugation plates with non-targeting control constructs grew on 5-FU selection plates (Fig. 2b), underlining the significant contribution of SpCas9 targeting to the high efficiency of the developed HR-SpCas9 counter selection tool in *R. sphaeroides*.

Prerequisite for extensive metabolic engineering studies with the HR-SpCas9 tool in *R. sphaeroides* is the performance of rapid iterative genome editing cycles. This demands the curing of constructed mutants from the SpCas9-harbouring editing plasmids via an easy and time-effective way. For this purpose, one of the previously constructed *Δupp* mutants was grown in LB broth medium without antibiotic for two iterative inoculation cycles of 24 h each. After plating and incubating the final culture on LB agar plates without antibiotic for 2 days, 5 colonies were selected and re-streaked on LB agar plates with and without antibiotic (kanamycin). Colonies that grew only on the agar plate without antibiotic were subjected to colony PCR with plasmid specific primers and the plasmid loss was verified (Additional file 1: Figure S4). The cured *R. sphaeroides* Δ*upp* strain was further employed for the expansion of the SpCas9-based toolbox.

### Homologous recombination-SpCas9 counter selection for gene insertions and single nucleotide substitutions

**Fig. 3:**
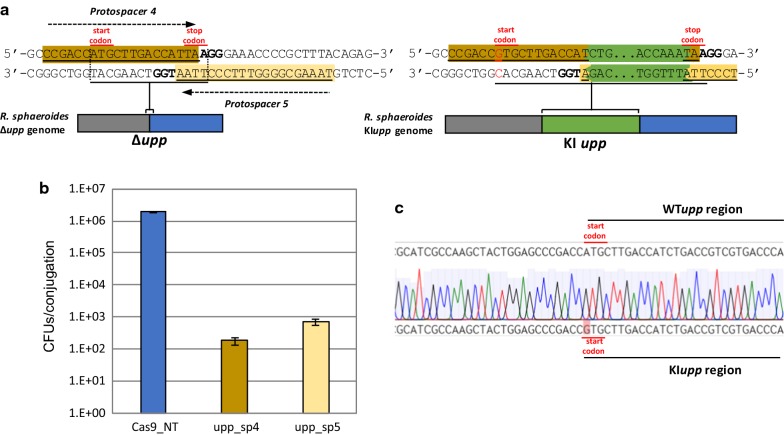
Insertion of the gene *upp* in a *Δupp* locus with single point mutation. **a** Representation of the *Δupp* locus before (left) and after (right) knock-in of the *upp* gene. The protospacers recognized by spacers sp4 and sp5 are underlined and highlighted with dark and light brown, respectively, and their PAM is in bold. The 1 kb flanking sites surrounding *Δupp* are in grey and blue, while the inserted *upp* sequence is in green; after introduction of the *upp* sequence, the protospacers sequences are disrupted, and the starting codon ATG is replaced by GTG via a 1 bp substitution (red). **b** Colony forming units (CFU) obtained after conjugation with pBBR_Cas9 plasmids (pBBR_Cas9_KIupp1000HR_sp4 and pBBR_Cas9_KIupp1000HR) harbouring different Δ*upp* targeting spacers (sp4 and sp5) and 1 kb HR templates for the knock-in of the *upp* gene accompanied by single nucleotide substitution; the plasmid with the non-targeting spacer (pBBR_Cas9_NT) was used as conjugation control. **c** Sequence verification of the desired *upp* insertion and single nucleotide substitution, by sanger sequencing

To further expand the genome editing toolbox of *R. sphaeroides*, we proceeded with the development of a plasmid-based HR-SpCas9 counter selection knock-in (KI) system. As a proof of principle, we designed a system for reinsertion of the *upp* gene in the genome of the previously constructed *Δupp* mutant strain. We selected two spacers (sp4 and sp5, Additional file 1: Table S5) for the editing plasmids, aiming to target the remaining fraction of the *upp* gene in the genome of the Δ*upp* mutant (Fig. 3a). Moreover, we constructed HR templates containing the *upp* gene - with the “ATG” start codon substituted by the “GTG” as sequence verification marker-flanked by 1 kb upstream and downstream genomic regions (pBBR_Cas9_KIupp1000HR_sp4 and pBBR_Cas9_KIupp1000HR_sp5). Upon reinsertion of the *upp* gene into the Δ*upp* genome the corresponding protospacers would be disrupted, providing resistance from the SpCas9 targeting.

The two editing plasmids and the pBBR_Cas9_NT control plasmid were conjugated in the plasmid-cured *R. sphaeroides* Δ*upp* mutant and the experiment was performed in 3 biological replicates. As expected, a decrease in the number of surviving conjugants was observed for both editing plasmids compared to the non-targeting control plasmid (Fig. 3b). The *upp* genomic region from 38 to 40 colonies per construct and per conjugation was amplified by colony PCR with genome specific primers. The editing constructs resulted in low editing efficiencies; 16/118 screened colonies for spacer 4 and only 2/120 screened colonies for spacer 5 were KI mutants (Additional file 1: Figure S5A, B). All the mutants were subsequently sequence verified for the existence of the “GTG” start codon (Fig. 3c). This result shows that, unlike the *upp* deletion process, the efficiency of the *upp* knock-in process is much lower, at least for the selected target.

### Eliminating PHB accumulation in *R. sphaeroides*

**Fig. 4:**
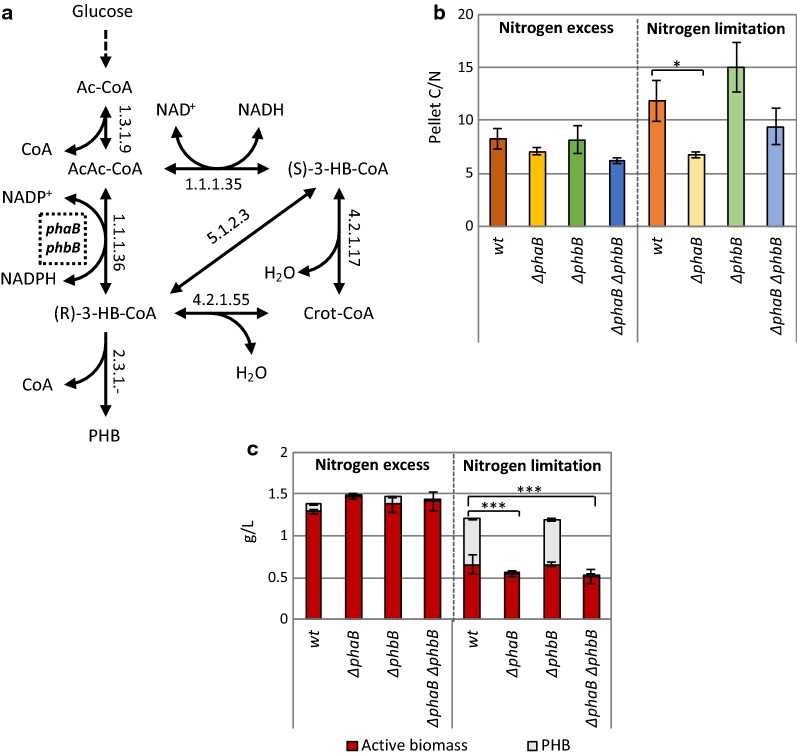
**a** The PHB biosynthetic pathway of *Rhodobacter sphaeroides.* The annotation is based on KEGG. Enzymatic conversions are indicated by their EC numbers. Double arrowed reactions describe reversible reactions (Δ_*r*_G′° > − 30 kJ/mol [48]). *Ac-CoA* acetyl-CoA; *AcAc-CoA* acetoacetyl-CoA; *(R)-3-HB* (R)-3-hydroxybutanoate, *(R)-3-HB-CoA* (R)-3-hydroxybutanoyl-CoA, *(S)-3-HB-CoA* (S)-3-hydroxybutanoyl-CoA, *Crot-CoA* crotonyl-CoA; PHB: poly-β-hydroxybutyrate. The interrupted square indicates the metabolic step that is hypothesized to be catalysed by *phaB* and/or *phbB*. **b** Effect of the *phaB* (RSP_0747) and the *phbB* (RSP_3963) knockout, as well as of the combined knockout, on the C/N ratios of the generated mutants in nitrogen excess and limiting conditions on a defined medium. Lower C/N ratio’s indicate less PHB accumulation (Student’s *t*-test, *P < 0.05). **c** Effect of the *phaB* (RSP_0747) and the *phbB* (RSP_3963) knockout, as well as of the combined knockout, on the acids production and the PHB accumulation in *Rhodobacter sphaeroides* (Student’s *t*-test, ***P < 0.001). Concentrations of active biomass, PHB, pyruvate and oxo-glutarate were measured after 24 h cultivation in Sistrom’s minimal medium supplied with 1.0 g/L or 0.25 g/L of NH_4_Cl (Nitrogen excess and nitrogen limited conditions, respectively). Error bars indicate standard deviations from at least two replicates

The intracellular accumulation of PHB in *R. sphaeroides* under nitrogen-limited culturing conditions acts as a carbon drain and a NADP^+^ regeneration mechanism, using acetoacetyl-CoA (AA-CoA) as precursor [6, 7, 46] (Fig. 4a). Hence, engineering a *R. sphaeroides* strain with reduced PHB production capacity could facilitate the increment of the intracellular NADPH and AA-CoA pools, and the channelling of the *R. sphaeroides* metabolism towards the production of alternative biotechnologically interesting molecules, such as hydrogen and terpenes.

AA-CoA reductases catalyse the NADPH-dependent conversion of AA-CoA into (R)3-hydroxybutyrate-CoA ((R) 3HB-CoA) which is the precursor of PHB. There are two predicted AA-CoA reductase genes in the genome of *R. sphaeroides*, namely *phaB* (RSP_0747) and *phbB* (RSP_3963), and the contribution of each corresponding enzyme to the AA-CoA reduction into (R) 3HB-CoA was the focus of this study. For this purpose, the *phaB* and *phbB* genes were separately and jointly knocked-out of the *R. sphaeroides* genome employing the previously described tool. More specifically, three editing plasmids were constructed and employed for the deletion of the *phaB* gene (namely pBBR_Cas9_ΔphaBHR_sp1, pBBR_Cas9_ΔphaBHR_sp2 and pBBR_Cas9_ΔphaBHR_sp3) and two for the knockout of the *phbB* gene (namely pBBR_Cas9_ΔphbBHR_sp1 and pBBR_Cas9_ΔphbBHR_sp2) via the introduction of premature stop codons in the sequence of the gene. We reasoned to not completely delete the *phbB* gene as it is located within an operon in the *R. sphaeroides* genome, unlike *phaB* which is located at the end of an operon (Additional file 1: Figure S6). The efficiency of the *phaB* deletion processes was 100% for constructs harbouring spacer sp1 (20/20 PCR screened colonies were clean *phaB* deletion mutants) and spacer sp2 (10/10 PCR screen colonies were clean *phaB* deletion mutants), but 0% efficiency for constructs harbouring spacer sp3 (0/13 PCR screened colonies were clean *phaB* deletion mutants) (Additional file 1: Figure S7). The efficiency of the *phbB* knockout processes was 100% when the pBBR_Cas9_ΔphbBHR_sp1 construct was employed (7/7 PCR screened colonies were clean *phbB* deletion mutants) and 0% when the pBBR_Cas9_ΔphbBHR_sp2 construct was employed (0/8 PCR screened colonies were clean *phbB* deletion mutants) (Additional file 1: Figure S8), confirming that the efficiency of the developed Cas9-based is spacer-dependent.

The C/N ratio of microbial biomass increases when PHB is produced. The C/N ratio of the wild type *R. sphaeroides* strain and the generated Δ*phaB*, Δ*phbB* and Δ*phaB*_Δ*phbB* strains was tested for PHB production after 48 h of cultivation under nitrogen excess and nitrogen-limited conditions. Analysis by TOC-L revealed no statistically relevant difference between the C/N ratios of the strains under nitrogen excess conditions (Fig. 4b, left panel). Under nitrogen limitation conditions, a substantial decrease was noticed in the C/N ratio of the Δ*phaB* strain as compared to the wild-type strain (Fig. 4b, Additional file 1: Table S6). This decrease indicated reduced carbon accumulation in the Δ*phaB* and Δ*phaB*_Δ*phbB* strains in comparison to the wild-type strain, which could be attributed to reduced PHB production.

In order to confirm the reduction in the PHB production, HPLC analysis was performed to quantify the intracellular PHB content of the wild-type, Δ*phaB*, Δ*phbB* and Δ*phaB*_Δ*phbB* strains. The analysis showed that the PHB content of the wild-type and Δ*phbB* strains can fluctuate between 40 and 70% (w/w) of the dry cellular weight (DCW), which is consistent with previously reported results [7, 47]. The PHB concentrations in the Δ*phaB* and Δ*phaB*_Δ*phbB* strains were comparable and more than 99% lower compared to the PHB concentrations of the wild type and Δ*phbB* strains. The active biomass of wild-type and mutant strains was calculated based on the TOC-L data (Fig. 4c, Additional file 1: Table S6). Only a minor reduction in the active biomass of the Δ*phaB* and Δ*phaB*_Δ*phbB* strains, as compared to the active biomass of the wild-type strain, was observed.

## Conclusions

In our work we developed a highly active SpCas9-based DNA targeting system for the biotechnologically interesting bacterium *R. sphaeroides*. We further combined the SpCas9 DNA targeting activity with the native homologous recombination (HR) mechanism of *R. sphaeroides* for the development of an efficient HR-SpCas9 counter selection-based genome editing toolbox for gene knock-outs, knock-ins and single nucleotide substitutions. Hence, the developed toolbox can substantially accelerate fundamental and metabolic engineering studies on *R. sphaeroides,* e.g. on membrane and photosynthetic reaction centre proteins, as well as metabolic engineering studies for improved hydrogen production and terpene synthesis, as already have been achieved for other microorganisms with developed SpCas9 toolboxes. Finally, we employed the developed toolbox to elucidate the genetic background of PHB production in *R. sphaeroides* and we constructed mutant strains with dramatically reduced PHB production capacity and almost unaffected growth. Hence, the here constructed *R. sphaeroides* mutant strains can be the basis for further engineering towards the enhanced production of alternative biotechnologically interesting molecules, such as hydrogen and terpenes.

## Supplementary information

**Additional file 1**. Additional figures and tables.

## Data Availability

The datasets supporting the conclusions of this article are included within the article (and its additional file).

## Abbreviations

PHB: poly-β-hydroxybutyrate
HR: homologous recombination
SCO: single crossover
*sacB*: levansucrase gene
CRISPR-Cas: Clustered Regularly Interspaced Short Palindromic Repeats-CRISPR associated proteins
DSB: double stranded DNA breaks
sgRNA: single guide RNA
PAM: protospacer adjacent motif
*upp*: uracyl-phosphoribosyltransferase gene
5-FU: 5-fluorouracil
AA-CoA: acetoacetyl-CoA
*phaB*: AA-CoA reductase
*phbB*: AA-CoA reductase

## References

[1] Imam S, Noguera DR, Donohue TJ (2013). Global insights into energetic and metabolic networks in *Rhodobacter sphaeroides*. BMC Syst Biol.

[2] Tabita RF (1995). The biochemistry and metabolic regulation of carbon metabolism and CO2 fixation in purple bacteria. Anoxygenic Photosynth Bact.

[3] Zannoni Davide, Schoepp-Cothenet Barbara, Hosler Jonathan (2009). Respiration and Respiratory Complexes. The Purple Phototrophic Bacteria.

[4] Nybo SE, Khan NE, Woolston BM, Curtis WR (2015). Metabolic engineering in chemolithoautotrophic hosts for the production of fuels and chemicals. Metab Eng.

[5] Kim M, Kim D, Cha J, Lee JK (2012). Effect of carbon and nitrogen sources on photo-fermentative H_2_ production associated with nitrogenase, uptake hydrogenase activity, and PHB accumulation in Rhodobacter sphaeroides KD131. Bioresour Technol.

[6] Ryu M-H, Hull NC, Gomelsky M (2014). Metabolic engineering of *Rhodobacter sphaeroides* for improved hydrogen production. Int J Hydrog Energy.

[7] Orsi E, Folch PL, Monje-López VT, Fernhout BM, Turcato A, Kengen SWM, Eggink G, Weusthuis RA (2019). Characterization of heterotrophic growth and sesquiterpene production by Rhodobacter sphaeroides on a defined medium. J Ind Microbiol Biotechnol.

[8] Lu W, Ye L, Lv X, Xie W, Gu J, Chen Z, Zhu Y, Li A, Yu H (2015). Identification and elimination of metabolic bottlenecks in the quinone modification pathway for enhanced coenzyme Q 10 production in *Rhodobacter sphaeroides*. Metab Eng.

[9] Hustede E, Steinbüchel A, Schlegel HG (1993). Relationship between the photoproduction of hydrogen and the accumulation of PHB in non-sulphur purple bacteria. Appl Microbiol Biotechnol.

[10] Porter SL, Wadhams GH, Armitage JP (2007). In vivo and in vitro analysis of the *Rhodobacter sphaeroides* chemotaxis signaling complexes. Methods Enzymol.

[11] Jaschke Paul R., Saer Rafael G., Noll Stephan, Beatty J. Thomas (2011). Modification of the Genome of Rhodobacter sphaeroides and Construction of Synthetic Operons. Methods in Enzymology.

[12] Su A, Chi S, Li Y, Tan S, Qiang S, Chen Z, Meng Y (2018). Metabolic redesign of *Rhodobacter sphaeroides* for lycopene production. J Agric Food Chem.

[13] Qiang S, Su AP, Li Y, Chen Z, Hu CY, Meng YH (2019). Elevated β-carotene synthesis by the engineered *Rhodobacter sphaeroides* with enhanced CrtY expression. J Agric Food Chem.

[14] Shimizu T, Teramoto H, Inui M (2019). Introduction of glyoxylate bypass increases hydrogen gas yield from acetate and L-glutamate in *Rhodobacter sphaeroides*. Appl Environ Microbiol.

[15] Kobayashi J, Kondo A (2019). Disruption of poly (3-hydroxyalkanoate) depolymerase gene and overexpression of three poly (3-hydroxybutyrate) biosynthetic genes improve poly (3-hydroxybutyrate) production from nitrogen rich medium by Rhodobacter sphaeroides. Microb Cell Fact.

[16] Zhu Y, Lu W, Ye L, Chen Z, Hu W, Wang C, Chen J, Yu H (2017). Enhanced synthesis of Coenzyme Q10 by reducing the competitive production of carotenoids in *Rhodobacter sphaeroides*. Biochem Eng J.

[17] Lee IH, Park J, Kho D, Kim MS, Lee J (2002). Reductive effect of H_2_ uptake and poly-β-hydroxybutyrate formation on nitrogenase-mediated H_2_ accumulation of *Rhodobacter sphaeroides* according to light intensity. Appl Microbiol Biotechnol.

[18] Mougiakos I, Bosma EF, Weenink K, Vossen EM, Goijvaerts K, Van Der Oost J, van Kranenburg R (2017). Efficient genome editing of a facultative thermophile using the mesophilic spCas9. ACS Synth Biol.

[19] Mougiakos I, Mohanraju P, Bosma EF, Vrouwe V, Finger Bou M, Naduthodi MIS, Gussak A, Brinkman RBL, Van Kranenburg R, Van Der Oost J (2017). Characterizing a thermostable Cas9 for bacterial genome editing and silencing. Nat Commun.

[20] Jinek M, Chylinski K, Fonfara I, Hauer M, Doudna JA, Charpentier E (2012). A programmable dual-RNA–guided DNA endonuclease in adaptive bacterial immunity. Science (80-).

[21] Hsu PD, Lander ES, Zhang F (2014). Development and applications of CRISPR-Cas9 for genome engineering. Cell.

[22] Selle K, Barrangou R (2015). Harnessing CRISPR-Cas systems for bacterial genome editing. Trends Microbiol.

[23] Doudna JA, Charpentier E (2014). The new frontier of genome engineering with CRISPR-Cas9. Science (80-).

[24] Mougiakos I, Bosma EF, Ganguly J, Van Der Oost J, Van Kranenburg R, Kranenburg R (2018). Hijacking CRISPR-Cas for high-throughput bacterial metabolic engineering: advances and prospects. Curr Opin Biotechnol.

[25] Gophna U, Allers T, Marchfelder A (2017). Finally, archaea get their CRISPR-Cas toolbox. Trends Microbiol.

[26] Stachler AE, Marchfelder A (2016). Gene repression in haloarchaea using the CRISPR (Clustered regularly interspaced short palindromic repeats)-Cas I-B system. J Biol Chem.

[27] Leenay RT, Beisel CL (2017). Deciphering, communicating, and engineering the CRISPR PAM. J Mol Biol.

[28] Arazoe T, Kondo A, Nishida K (2018). Targeted nucleotide editing technologies for microbial metabolic engineering. Biotechnol J.

[29] Bikard D, Jiang W, Samai P, Hochschild A, Zhang F, Marraffini LA (2013). Programmable repression and activation of bacterial gene expression using an engineered CRISPR-Cas system. Nucleic Acids Res.

[30] Dong C, Fontana J, Patel A, Carothers JM, Zalatan JG (2018). Synthetic CRISPR-Cas gene activators for transcriptional reprogramming in bacteria. Nat Commun.

[31] Zheng K, Wang Y, Li N, Jiang F-F, Wu C-X, Liu F, Chen H-C, Liu Z-F (2018). Highly efficient base editing in bacteria using a Cas9-cytidine deaminase fusion. Commun Biol.

[32] Banno S, Nishida K, Arazoe T, Mitsunobu H, Kondo A (2018). Deaminase-mediated multiplex genome editing in *Escherichia coli*. Nat Microbiol.

[33] Gaudelli NM, Komor AC, Rees HA, Packer MS, Badran AH, Bryson DI, Liu DR (2017). Programmable base editing of A T to G C in genomic DNA without DNA cleavage. Nature.

[34] Mougiakos I, Bosma EF, de Vos WM, van Kranenburg R, van der Oost J (2016). Next generation prokaryotic engineering: the CRISPR-Cas toolkit. Trends Biotechnol.

[35] Claassens NJ, Siliakus MF, Spaans SK, Creutzburg SCA, Nijsse B, Schaap PJ, Quax TEF, Van Der Oost J (2017). Improving heterologous membrane protein production in *Escherichia coli* by combining transcriptional tuning and codon usage algorithms. PLoS ONE.

[36] Waligórska M, Seifert K, Górecki K, Moritz M, Łaniecki M (2009). Kinetic model of hydrogen generation by Rhodobacter sphaeroides in the presence of NH_4_^+^ ions. J Appl Microbiol..

[37] Li H, Shen CR, Huang CH, Sung LY, Wu MY, Hu YC (2016). CRISPR-Cas9 for the genome engineering of cyanobacteria and succinate production. Metab Eng.

[38] Wendt KE, Ungerer J, Cobb RE, Zhao H, Pakrasi HB (2016). CRISPR/Cas9 mediated targeted mutagenesis of the fast growing cyanobacterium *Synechococcus elongatus* UTEX 2973. Microb Cell Fact.

[39] Jiang Y, Qian F, Yang J (2017). CRISPR-Cpf1 assisted genome editing of *Corynebacterium glutamicum*. Nat Commun.

[40] Huo J (2011) Design of a BioBrick TM compatible gene expression system for *Rhodobacter sphaeroides*. Utah State University.

[41] Cohen SS, Flaks JG, Barner HD, Loeb MR, Lichtenstein J (1958). The mode of action of 5-fluorouracil and its derivatives. Proc Natl Acad Sci USA.

[42] Singh V, Brecik M, Mukherjee R (2015). The complex mechanism of antimycobacterial action of 5-fluorouracil. Chem Biol.

[43] Selle K, Klaenhammer TR, Barrangou R (2015). CRISPR-based screening of genomic island excision events in bacteria. Proc Natl Acad Sci USA.

[44] Vercoe RB, Chang JT, Dy RL, Taylor C, Gristwood T, Clulow JS, Richter C, Przybilski R, Pitman AR, Fineran PC (2013). Cytotoxic chromosomal targeting by CRISPR/Cas systems can reshape bacterial genomes and expel or remodel pathogenicity islands. PLoS Genet.

[45] Cui L, Bikard D (2016). Consequences of Cas9 cleavage in the chromosome of *Escherichia coli*. Nucleic Acids Res.

[46] Hong SH, Park SJ, Moon SY, Park JP, Lee SY (2003). In silico prediction and validation of the importance of the Entner-Doudoroff pathway in poly(3-hydroxybutyrate) production by metabolically engineered *Escherichia coli*. Biotechnol Bioeng.

[47] Brandl H, Gross RA, Lenz RW, Lloyd R, Fuller RC (1991). The accumulation of poly(3-hydroxyalkanoates) in *Rhodobacter sphaeroides*. Arch Microbiol.

[48] Flamholz A, Noor E, Bar-Even A, Milo R (2012). eQuilibrator—the biochemical thermodynamics calculator. Nucleic Acids Res.

