# Supplementary material for: Efficient Cas9-based genome editing of Rhodobacter sphaeroides for metabolic engineering
Source: Microb Cell Fact. 2019 Nov 25;18:204. doi: 10.1186/s12934-019-1255-1 (PMC6876111; doi:10.1186/s12934-019-1255-1)
Supplement: Supplementary file 1 — Additional file 1. Additional figures and tables. [file 12934_2019_1255_MOESM1_ESM.docx]

Efficient Cas9-based genome editing of *Rhodobacter sphaeroides* for metabolic engineering

Ioannis Mougiakos ^1^*, Enrico Orsi ^2^*, Mohammad Rifqi Ghiffary^1,2^, Wilbert Post^1,2^, Alberto de Maria^1,2^, Belén Adiego-Perez^1,2^, Servé W.M. Kengen^1^, Ruud A. Weusthuis^2^**, John van der Oost^1^**

# Additional Information


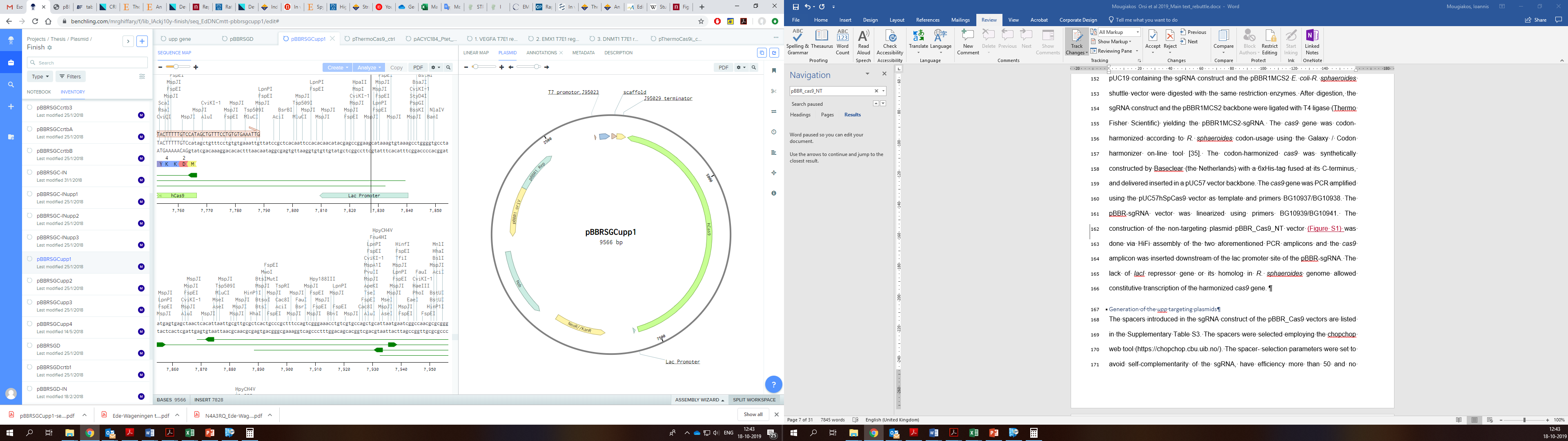


J95029 terminator

J95029 promoter

sgRNA

Lac promoter

**pBBR_Cas9_NT**

**Figure S1.** Graphical representation of the pBBR_Cas9 _NT plasmid map.

2x

RÄ wash


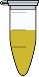

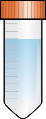

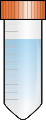

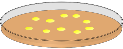

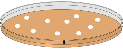

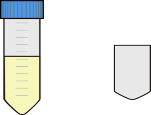


*R. sphaeroides*

*E. coli* S17

48 h

(RÄ)

o/n

LB+Kan50


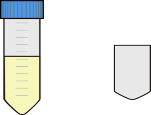


2 h 2xYT LB+Kan50


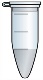


1 mL


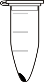

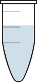


concentrate into 100 µL


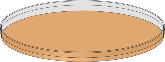


100 µL

onto filter on PY plate

6 h at 28^o^C


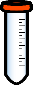


harvest

with 2 mL RÄ medium


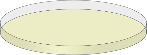


3 days at 30^o^C

100 µL

RÄ agar

+Kan50

24 h

(RÄ)

(1:1) combined in 2mL

**Figure S2.** Schematic overview of the di-parental conjugation protocol used: detailed description of the protocol can be found in the Material and Methods section.

**Figure S3.** 1% agarose electrophoresis showing the PCR amplified *upp* genomic regions from 5 surviving colonies conjugated with the pBBR_Cas9_sp3 plasmid; all the amplicons showed the wild-type size

**Figure S4.** Curing from the plasmid after successful conjugation. A) Schematic overview of the workflow: mutants from *R. sphaeroides* were transferred twice to liquid medium without antibiotic and incubated over-night. Subsequently, the liquid culture was spread on LB plates. Once colonies formed, they were transferred to LB plates with and without antibiotic. B) The colonies that grew only on the plate without antibiotic were PCR amplified with primers annealing to the *cas9* gene in the vector backbone using primers set BG10937/BG10938 (P: plasmid control, 1 and 2: curated *R. sphaeroides* mutants).

**Figure S5.** 1% agarose electrophoresis showing the 3 rounds of colony PCRs after the conjugations of the *R. sphaeroides Δupp* strain with the plasmids for *upp* insertion. A) pBBR_Cas9_KIupp1000HR_sp4. B) pBBR_Cas9_KIupp1000HR_sp5. C) Schematic representation of the amplicons generated via colony PCR: the Δ*upp* gene leads to an amplicon of 2053bp (left), while a restored *upp* gene (green) generates an amplicon of 2903bp. The homology regions are highlighted in yellow.

**Figure S6.** Genomic landscape of the *phaB* and *phbB* genes within the *R. sphaeroides genome.*


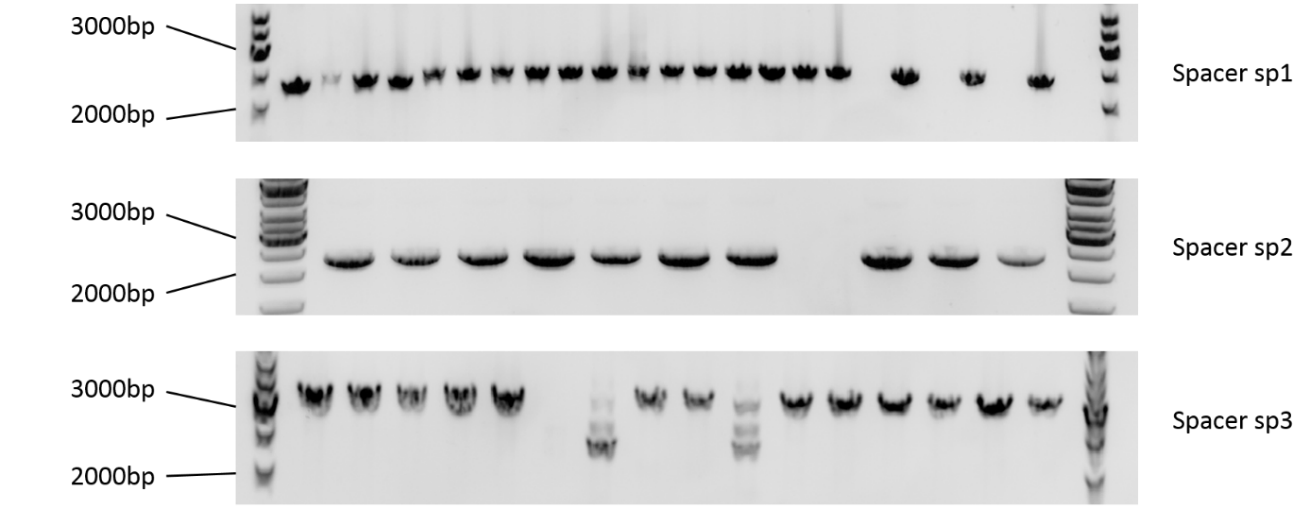


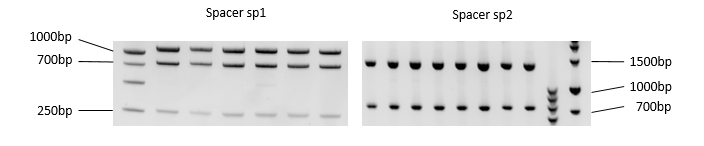
**Figure S7.** Figure S4. 1.2% agarose electrophoresis showing the colony PCRs after the conjugation of the WT strain of *R. sphaeroides* with the plasmids for knockout of *phaB*. Upper panel: pBBR_Cas9_ΔphaBHR_sp1, middle panel: pBBR_Cas9_ΔphaBHR_sp2, lower panel: pBBR_Cas9_ΔphaBHR_sp3. Wildtype *phaB* amplification yields an amplicon of 2947bp whereas Δ*phaB* would result in a 2224bp fragment.

**Figure S8.** Figure S5. 1.2% agarose electrophoresis showing restriction digestion by *Eco*RI of colony PCRs after the conjugation of the WT strain of *R. sphaeroides* with the plasmids for knockout of *phbB*. Left panel: pBBR_Cas9_ΔphbBHR_sp1, right panel: pBBR_Cas9_ΔphaBHR_sp2. Wild-type *phbB* digestion would yield fragments of 786 and 1383bp whereas Δ*phbB* would lead to fragments of 261, 786 and 1099bp.

**Table S1**. List of plasmids used in this study

| **Plasmid** | **Description** | **Source** |
| --- | --- | --- |
| pUC57hCas9 | pUC57 vector containing DNA encoding the *cas9* gene harmonized for expression in *Rhodobacter sphaeroides* (*cas9_h_*) | Baseclear |
| pBBR1MCS2-sgRNA | pBBR1MCS2-sgRNA derivative. Contains the BBa_J95023 promoter followed by two BsaI restriction sites, the sgRNA scaffold sequence and the BBa_J95029 terminator | Baseclear, Huo et. al, 2011, this study |
| pBBR1MCS2_Cas9_NT | Broad host range plasmid (KanR) containing the *cas9_h_* under the control of the constitutive P_lac_ promoter and the non-targeting sgRNA under the control of the BBa_J95023 promoter and the BBa_J95029 terminator | this study |
| pBBR1MCS2_Cas9_sp1 | pBBR1MCS2_Cas9_NT derivative, containing sgRNA with spacer 1 for targeting the *upp* gene | this study |
| pBBR1MCS2_Cas9_sp2 | pBBR1MCS2_Cas9_NT derivative, containing sgRNA with spacer 2 for targeting the *upp* gene | this study |
| pBBR1MCS2_Cas9_sp3 | pBBR1MCS2_Cas9_NT derivative, containing sgRNA with spacer 3 for targeting the *upp* gene | this study |
| pBBR_Cas9_Δupp500HR_NT | pBBR1MCS2_Cas9_NT derivative containing a non-targeting sgRNA and the genetic sequences 500bp upstream and downstream of the *upp* gene -fused in one fragment- serving as homologous recombination template | this study |
| pBBR_Cas9_Δupp1000HR_NT | pBBR1MCS2_Cas9_NT derivative containing a non-targeting sgRNA and the genetic sequences 1kb upstream and downstream of the *upp* gene -fused in one fragment- serving as homologous recombination template | this study |
| pBBR_Cas9_Δupp500HR_sp2 | pBBR1MCS2_Cas9_NT derivative containing a sgRNA with spacer 2 and the genetic sequences 500bp upstream and downstream of the *upp* gene -fused in one fragment- serving as homologous recombination template | this study |
| pBBR_Cas9_Δupp1000HR_sp2 | pBBR1MCS2_Cas9_NT derivative containing a sgRNA with spacer 2 and the genetic sequences 1kb upstream and downstream of the *upp* gene -fused in one fragment- serving as homologous recombination template | this study |
| pBBR_Cas9_KIupp500HR_sp4 | pBBR1MCS2_Cas9_NT derivative containing a sgRNA with spacer 4 for targeting the *Δupp* genomic locus previously and 500bp-long flanking sites for knock-in of the *upp* gene back in the *Δupp* strain via homologous recombination | this study |
| pBBR_Cas9_KIupp500HR_sp5 | pBBR1MCS2_Cas9_NT derivative containing a sgRNA with spacer 5 for targeting the *Δupp* genomic locus previously and 500bp-long flanking sites for knock-in of the *upp* gene back in the *Δupp* strain via homologous recombination | this study |
| pBBR_Cas9_KIupp1000HR_sp4 | pBBR1MCS2_Cas9_NT derivative containing a sgRNA with spacer 4 for targeting the *Δupp* genomic locus previously and 1kb-long flanking sites for knock-in of the *upp* gene back in the *Δupp* strain via homologous recombination | this study |
| pBBR_Cas9_KIupp1000HR_sp5 | pBBR1MCS2_Cas9_NT derivative containing a sgRNA with spacer 5 for targeting the *Δupp* genomic locus previously and 1kb-long flanking sites for knock-in of the *upp* gene back in the *Δupp* strain via homologous recombination | this study |
| pBBR_Cas9_ΔphaBHR_sp1 | pBBR1MCS2_Cas9_NT derivative containing a sgRNA with spacer 1 targeting the *phaB* gene the genetic sequences 1kb upstream and downstream of the *phaB* gene -fused in one fragment- serving as homologous recombination template | this study |
| pBBR_Cas9_ΔphaBHR_sp2 | pBBR1MCS2_Cas9_NT derivative containing a sgRNA with spacer 2 targeting the *phaB* gene the genetic sequences 1kb upstream and downstream of the *phaB* gene -fused in one fragment- serving as homologous recombination template | this study |
| pBBR_Cas9_ΔphaBHR_sp3 | pBBR1MCS2_Cas9_NT derivative containing a sgRNA with spacer 3 targeting the *phaB* gene the genetic sequences 1kb upstream and downstream of the *phaB* gene -fused in one fragment- serving as homologous recombination template | this study |
| pBBR_Cas9_ΔphbBHR_sp1 | pBBR1MCS2_Cas9_NT derivative containing a sgRNA with spacer 1 targeting the *phbB* gene the genetic sequences 1kb upstream and downstream of the *phbB* gene -fused in one fragment- serving as homologous recombination template | this study |
| pBBR_Cas9_ΔphbBHR_sp2 | pBBR1MCS2_Cas9_NT derivative containing a sgRNA with spacer 2 targeting the *phbB* gene the genetic sequences 1kb upstream and downstream of the *phbB* gene -fused in one fragment- serving as homologous recombination template | this study |
|  |  |  |

**Table S2**. List of primers used in this study

|  | **Oligo** | **Sequence** | **Description** |
| --- | --- | --- | --- |
| Ubiquitin PCR | P3-Ec_ubi1 | GATAGCGTCCTAACGGCGTT | FW for ubiquitin PCR to check post-conjugational *E. coli* contamination. Anneals to *E .coli* genome |
|  | P4-Ec_ubi2 | GGGTTTGTCGAGCAGAATGA | RV for ubiquitin PCR to check post-conjugational *E. coli* contamination. Anneals to *E. coli* genome |
|  | P5-Rs_ubi105F | GAACCCCTGCCGGCTGGATTACA | FW for ubiquitin PCR to check post-conjugational *E. coli* contamination. Anneals to *R. sphaeroides* genome |
|  | P6-Rs_ubi501R | CATGGCGAAGCTCTTGGGATTG | RV for ubiquitin PCR to check post-conjugational *E. coli* contamination. Anneals to *R. sphaeroides* genome |
| RT-PCR Cas9 | BG11112 | GCCCGTTCTCGAGTTCG | FW to check Cas9 transcription in pBBR_Cas9_NT |
|  | BG11115 | GAATCGATCCTCCCCAAGAG | RV to check Cas9 transcription in pBBR_Cas9_NT |
| Non-targeting: pBBR_Cas9_NT | BG10937 | GACAAAAAGTACTCGATTGGGC | FW for amplifying *cas9_h_* from pUC57 |
|  | BG10938 | CTAATGGTGATGGTGATGGTGATC | RV for amplifying *cas9_h_* from pUC57 |
|  | BG10939 | GCCCAATCGAGTACTTTTTGTCCATAGCTGTTTCCTGTGTGAAATTG | FW for linearizing pBBR1MCS2-sgRNA with homologous overhang for Gibson assembly with *cas9_h_* |
|  | BG10941 | TCACCATCACCATCACCATTAGGAGGTCGACGGTGATTGATTGAG | RV for linearizing pBBR1MCS2-sgRNA with homologous overhang for Gibson assembly with *cas9_h_* |
| *upp targeting* | BG11415 | ACCTTAACAAGCTCGTCGACGAC | FW for linearizing the conserved parts of *cas9_h_* and backbone of pBBR_Cas9_NT |
|  | BG11416 | GCTATCTGGACAAGGGAAAACGC | RV for linearizing the conserved parts of *cas9_h_* and backbone of pBBR_Cas9_NT |
|  | BG11412 | GTCGTCGACGAGCTTGTTAAGGT | RV conserved for amplifying part of the *cas9_h_* gene and include the spacer of interest for *upp* in the sgRNA (provided by FW) |
|  | BG11411 | CGGCGTCTGGATCGTCGTCGGTTTTAGAGCTAGAAATAGCAAGTTAAAATAAGGCTAGTC | FW to combine with BG11412 for amplifying the sgRNA including the spacer 3 for *upp* and part of *cas9_h_* gene |
|  | BG11486 | CACCTTCGCATAATATTGCAGTTTTAGAGCTAGAAATAGCAAGTTAAAATAAGGCTAGTC | FW to combine with BG11412 for amplifying the sgRNA including the spacer 1 for *upp* and part of *cas9_h_* gene |
|  | BG11487 | ATCGATCTCCTGAAGGAGAAGTTTTAGAGCTAGAAATAGCAAGTTAAAATAAGGCTAGTC | FW to combine with BG11412 for amplifying the sgRNA including the spacer 2 for *upp* and part of *cas9_h_* gene |
|  | BG11413 | GCGTTTTCCCTTGTCCAGATAGC | FW conserved for amplifying the conserved part of the backbone and include the spacer of interest for *upp* in the sgRNA (provided by RV) |
|  | BG11414 | CGACGACGATCCAGACGCCGAACCAGCGATCCCGTCCG | RV to combine with BG11413 for amplifying the sgRNA including the spacer 3 for *upp* and part of pBBR1MCS2-sgRNA backbone |
|  | BG11488 | TGCAATATTATGCGAAGGTGAACCAGCGATCCCGTCCG | RV to combine with BG11413 for amplifying the sgRNA including the spacer 1 for *upp* and part of pBBR1MCS2-sgRNA backbone |
|  | BG11489 | TTCTCCTTCAGGAGATCGATAACCAGCGATCCCGTCCG | RV to combine with BG11413 for amplifying the sgRNA including the spacer 2 for *upp* and part of pBBR1MCS2-sgRNA backbone |
| *upp* knock-out | BG11886 | GTGCGGGCCTCTTCGCTATTA | FW to amplify pBBR_Cas9NT including the sgRNA with non-targeting spacer and the *cas9_h_* gene |
|  | BG11182 | CCATGTCGGCAGAATGCTTAATG | RV to amplify pBBR_Cas9NT including the sgRNA with non-targeting spacer and the *cas9_h_* gene |
|  | BG11887 | CATTAAGCATTCTGCCGACATGG | FW to amplify pBBR_Cas9NT including the backbone |
|  | BG11888 | GCCTGAATGGCGAATGGAAATTGTAA | RV to amplify pBBR_Cas9NT including the backbone |
|  | BG11866 | CTCTGTAAAGCGGGGTTTCCCTTAATGGTCAAGCATGGTCGGGC | FW for 500/1000 bp amplification of the first homology region of *upp.* It contains overhangs with BG11871 |
|  | BG11867 | TAATAGCGAAGAGGCCCGCACCGACGAGACGGTGGGCAA | RV for 500 bp amplification of the homology region of *upp.* |
|  | BG11869 | TTACAATTTCCATTCGCCATTCAGGCTTTCCACGTCTTCGCCGG | FW for 500 bp amplification of the second homology region of *upp.* |
|  | BG11871 | TAAGGGAAACCCCGCTTTACAGAG | RV for 500/1000 bp amplification of the second homology region of *upp.* It contains overhangs with BG11866 |
|  | BG11868 | TAATAGCGAAGAGGCCCGCACCGCATCTGGGCCTCTCCAA | RV for 1000 bp amplification of the first homology region of *upp.* |
|  | BG11870 | TTACAATTTCCATTCGCCATTCAGGCTGTCTCTAGCGGAGGAATGCGT | FW for 1000 bp amplification of the second homology region of *upp.* |
| *upp* knock-in | BG12347 | GATGGTCAAGCACGGTCGGG | FW for *upp* flanking site 1 amplification, including overhang for BG12348 and point mutation for the start ATG codon |
|  | BG12348 | CCCGACCGTGCTTGACCATC | RV for *upp* flanking site 1 amplification, including overhang for BG12347 and point mutation for the start ATG codon |
|  | BG12908 | TAATGGTCAAGCATGGTCGGAACCAGCGATCCCGTCCG | RV to combine with BG11886 for amplifying the sgRNA including the spacer 4 for *Δupp* and part of pBBR1MCS2-sgRNA backbone |
|  | BG12907 | CCGACCATGCTTGACCATTAGTTTTAGAGCTAGAAATAGCAAGTTAAAATAAGGCTAGTC | FW to combine with BG11182 for amplifying the sgRNA including the spacer 4 for *Δupp* and part of *hCas9* gene |
|  | BG12910 | TTAAGGGAAACCCCGCTTTAAACCAGCGATCCCGTCCG | RV to combine with BG11886 for amplifying the sgRNA including the spacer 5 for *Δupp* and part of pBBR1MCS2-sgRNA backbone |
|  | BG12909 | TAAAGCGGGGTTTCCCTTAAGTTTTAGAGCTAGAAATAGCAAGTTAAAATAAGGCTAGTC | FW to combine with BG11182 for amplifying the sgRNA including the spacer 5 for *Δupp* and part of *cas9_h_* gene |
| *phaB and phbB* knock-out | P301 | CCATGTCGGCAGAATGCTTAATG | RV to amplify pBBR_Cas9NT including the sgRNA with non-targeting spacer and the *cas9_h_* gene |
|  | P302 | CATTAAGCATTCTGCCGACATGG | FW to amplify pBBR_Cas9NT including the backbone |
|  | P303 | GCCTGAATGGCGAATGGAAATTGTAA | RV to amplify pBBR_Cas9NT including the backbone |
|  | P304 | GTGCGGGCCTCTTCGCTATTA | FW to amplify pBBR_Cas9NT including the sgRNA with non-targeting spacer and the *cas9_h_* gene |
|  | P379 | TAATAGCGAAGAGGCCCGCAC**ATGTCCAGCTGGGCGAC** | RV for *phaB* flanking site 1, amplification, with overhang for the backbone |
|  | P380 | TTTTCAGGATGAGAAGATCC**GATCCCTCCTTCAAATATCCGC** | FW for *phaB* flanking site 1, amplification, with overhang for flanking site 2 |
|  | P381 | GGATATTTGAAGGAGGGATC**GGATCTTCTCATCCTGAAAACCA** | RV for *phaB* flanking site 1, amplification, with overhang for flanking site 1 |
|  | P382 | TTACAATTTCCATTCGCCATTCAGGCGAGCCTCTGTCTCGC | FW for *phaB* flanking site 1, amplification, with overhang for the backbone |
|  | P383 | GACCTACAAATGGTCCGTCG**GTTTTAGAGCTAGAAATAGCAAGTTAAAATAAGGCTAGTC** | FW for sgRNA sp1 , spCas9 and backbone |
|  | P384 | CGACGGACCATTTGTAGGTC**AACCAGCGATCCCGTCCG** | RV for sgRNA sp1 , backbone, overhang for flanking site 1 |
|  | P385 | CGGGCAGGCGAACTATTCGG**GTTTTAGAGCTAGAAATAGCAAGTTAAAATAAGGCTAGTC** | FW for sgRNA sp2 , spCas9 and backbone |
|  | P386 | CCGAATAGTTCGCCTGCCCG**AACCAGCGATCCCGTCCG** | RV for sgRNA sp2 , backbone, overhang for flanking site 1 |
|  | P387 | CAATCTCCTCCGGTTCGCCGGTTTTAGAGCTAGAAATAGCAAGTTAAAATAAGGCTAGTC | FW for sgRNA sp3 , spCas9 and backbone |
|  | P388 | CGGCGAACCGGAGGAGATTGAACCAGCGATCCCGTCCG | RV for sgRNA sp3 , backbone, overhang for flanking site 1 |
|  | P413 | TAATAGCGAAGAGGCCCGCACAGGAGAATATCGAGCTCGGC | RV for *phbB* flanking site 1, amplification, with overhang for the backbone |
|  | P414 | TAGGAATTCCTACTACTACTACACGGTGATGCCGAAGG | FW for *phbB* flanking site 1, amplification, with overhang for flanking site 2 |
|  | P415 | GTGTAGTAGTAGTAGGAATTCCTACGATGCGGCGCTGGCC | RV for *phbB* flanking site 1, amplification, with overhang for flanking site 1 |
|  | P416 | TTACAATTTCCATTCGCCATTCAGGCTTGCGCGCCCGGGTCTCA | FW for *phbB* flanking site 1, amplification, with overhang for the backbone |
|  | P417 | TCGGCAGCTGCTTCGGACCGAACCAGCGATCCCGTCCG | FW for sgRNA sp1 , spCas9 and backbone |
|  | P418 | CGGTCCGAAGCAGCTGCCGAGTTTTAGAGCTAGAAATAGCAAGTTAAAATAAGGCTAGTC | RV for sgRNA sp1 , backbone, overhang for flanking site 1 |
|  | P419 | CGGTCCGAAGCAGCTGCCGAAACCAGCGATCCCGTCCG | FW for sgRNA sp2 , spCas9 and backbone |
|  | P420 | TCGGCAGCTGCTTCGGACCGGTTTTAGAGCTAGAAATAGCAAGTTAAAATAAGGCTAGTC | RV for sgRNA sp2 , backbone, overhang for flanking site 1 |
| sequencing | BG12037 | GGCCGAAGGGCTGCATC | for Δ*upp* sequencing |
|  | BG12038 | CATGGCAACGATCCCACCTT | for Δ*upp* sequencing |
|  | P409 | AGCATCAACCAGGTCTGCGG | For sequencing *phaB* knockout |
|  | P410 | ACAACGGCATCCCGACGA | For sequencing *phaB* knockout |
|  | P423 | CGAGGACCGGCGCATCTT | For Δ*phbB* sequencing |
|  | P424 | CGGGCATCCTGTTCCTGATGT | For Δ*phbB* sequencing |
|  |  |  |  |

**Table S3**. The sequence of the sgRNA expressing module (promoter BBa_J95023, sgRNA scaffold and BBa_J95029 terminator) in FASTA format

>sgRNA-module
TCGTCTCTTCGTCATTTTTCCTCTTGCGGGTTTTTTTGCGGTTCCCTAGATAGCGCCTCACCGAAGCGGAACGGCGACGGTGACGGGGTTGAGAGGCGGCGGTGCTGCCTTGAGGCTTTCGGAAATCTGGAAGATGAGGCGGACGGGATCGCTGGTTNNNNNNNNNNNNNNNNNNNNGTTTTAGAGCTAGAAATAGCAAGTTAAAATAAGGCTAGTCCGTTATCAACTTGAAAAAGTGGCACCGAGTCGGTGCTTTGATCCGGTGGATGACCTTTTGAATGACCTTTAATAGATTATATTACTAATTAATTGGGGACCCTAGAGGTCCCCTTTTTTATTTTAAAAATTTTTTCACAAAACGGTTTACAAGCATAAAGCTTGCTCAATCAATCACC

**Table S4**. The sequence of the codon harmonised *cas9* gene (*cas9_h_*)for *R. sphaeroides* in FASTA format

>hcas9
ATGGACAAAAAGTACTCGATTGGGCTCGACATCGGGACCAACTCCGTTGGGTGGGCTGTTATCACCGACGAGTACAAAGTCCCTTCGAAGAAATTTAAAGTCTTGGGGAACACCGATCGTCATAGCATCAAGAAGAACCTTATTGGAGCGCTTCTCTTCGATAGCGGGGAAACCGCTGAGGCTACCCGGTTGAAGCGAACCGCGCGGAGGAGGTACACCCGGCGAAAAAACCGGATCTGCTACCTTCAGGAAATCTTCTCGAACGAAATGGCTAAGGTTGACGACAGCTTTTTCCACCGTCTTGAGGAATCGTTCCTTGTTGAGGAGGATAAAAAACACGAGCGGCACCCCATCTTCGGGAACATTGTTGACGAGGTCGCGTACCACGAAAAGTACCCCACCATCTACCACTTGCGTAAGAAGCTTGTTGACTCGACCGACAAGGCTGACCTTCGTCTCATCTACCTTGCACTCGCTCACATGATCAAATTCCGGGGGCACTTCCTTATCGAAGGGGACCTCAACCCCGACAACAGCGACGTTGATAAGCTTTTCATCCAGCTTGTTCAGACGTACAACCAGCTCTTCGAGGAGAACCCCATCAACGCTAGCGGGGTTGACGCGAAGGCTATCCTTTCGGCTCGTCTTAGCAAGTCGAGGCGTCTCGAGAACTTGATCGCGCAGTTGCCTGGGGAAAAAAAGAACGGGCTCTTCGGAAACTTGATCGCGCTTTCGCTTGGGCTTACGCCCAACTTCAAGTCGAACTTCGACCTTGCTGAGGACGCGAAGCTCCAGCTTTCGAAGGACACCTACGACGACGACCTCGACAACCTCCTTGCTCAGATCGGGGACCAGTACGCGGACCTTTTCCTTGCTGCGAAAAACCTCTCGGACGCGATCCTCCTTTCGGACATCCTTAGGGTTAACACCGAGATTACCAAAGCGCCTCTTTCGGCGTCGATGATCAAGCGTTACGACGAGCACCACCAGGATCTTACCCTTCTCAAGGCGCTCGTCCGTCAGCAGCTTCCCGAGAAATACAAGGAGATCTTCTTCGACCAGTCGAAGAACGGGTACGCTGGGTACATCGACGGAGGGGCGTCCCAGGAGGAGTTCTACAAGTTCATCAAGCCCATCCTCGAGAAGATGGACGGGACCGAAGAGCTCCTTGTTAAGCTTAACCGGGAGGACCTTTTGCGTAAACAGCGAACGTTCGATAACGGGTCGATCCCTCACCAGATCCATCTTGGGGAATTGCACGCGATCCTTAGGAGGCAGGAGGATTTCTACCCCTTCCTCAAGGATAACCGGGAAAAAATCGAGAAGATCCTTACCTTCCGTATCCCCTACTACGTCGGGCCCCTTGCTCGGGGGAACAGCCGGTTCGCTTGGATGACCCGAAAATCGGAGGAGACCATCACGCCCTGGAACTTCGAGGAGGTCGTTGACAAGGGGGCGTCGGCGCAGTCGTTCATCGAGCGTATGACCAACTTCGACAAGAACCTTCCCAACGAGAAGGTTCTTCCCAAGCACAGCCTTCTTTACGAATACTTCACGGTCTACAACGAGCTTACCAAAGTTAAGTACGTCACCGAGGGGATGCGTAAGCCCGCTTTCCTTTCGGGGGAGCAGAAAAAGGCAATCGTCGACCTCTTGTTTAAGACCAACCGTAAGGTTACGGTCAAACAGCTCAAGGAGGACTACTTTAAGAAGATTGAGTGCTTCGACAGCGTCGAGATCTCGGGGGTCGAGGACAGGTTCAACGCGTCGCTCGGGACGTACCACGACCTTCTTAAGATCATCAAGGACAAGGACTTCCTTGACAACGAGGAGAACGAGGACATCCTCGAAGACATCGTCCTCACCCTTACGCTCTTCGAGGACAGGGAAATGATCGAAGAGAGGCTTAAGACCTACGCGCATTTGTTCGACGACAAAGTTATGAAGCAGCTTAAGCGGCGTCGGTACACCGGGTGGGGGCGGCTTTCGCGTAAGCTTATCAACGGGATCAGGGACAAACAGTCGGGGAAGACCATTCTCGACTTCCTTAAGTCGGACGGGTTCGCAAACCGTAACTTCATGCAGTTGATCCACGACGACAGCCTTACCTTCAAGGAGGATATCCAGAAGGCTCAGGTTTCGGGGCAGGGGGACAGCCTCCACGAGCACATCGCTAACCTCGCGGGGTCCCCCGCGATCAAGAAGGGGATCCTCCAGACCGTTAAGGTCGTCGACGAGCTTGTTAAGGTTATGGGACGACACAAACCCGAGAACATCGTCATCGAGATGGCTCGGGAGAACCAGACCACCCAGAAAGGGCAGAAGAACTCCCGTGAACGGATGAAGCGTATCGAGGAGGGGATCAAGGAGCTCGGGAGCCAGATCCTTAAGGAACACCCCGTCGAGAACACCCAGCTTCAGAACGAGAAATTGTACTTGTACTACTTGCAGAACGGGAGGGATATGTACGTTGATCAGGAGCTCGACATCAACCGGCTCAGCGACTACGACGTTGACCATATCGTCCCCCAGAGCTTTCTTAAGGATGACTCGATTGATAACAAAGTTCTCACGCGGTCGGACAAGAACCGGGGGAAGTCCGACAACGTCCCCAGCGAGGAGGTTGTTAAGAAAATGAAGAACTACTGGAGGCAGCTTCTTAACGCAAAACTCATCACCCAGCGGAAATTCGACAACCTCACGAAGGCGGAGCGGGGGGGGCTTAGCGAGCTTGACAAGGCGGGGTTCATCAAGCGTCAGCTTGTCGAGACCCGTCAGATCACCAAACACGTTGCTCAGATCCTTGACAGCCGTATGAACACCAAGTACGACGAGAACGACAAGCTTATCCGTGAAGTCAAGGTTATCACGCTCAAGTCGAAGCTCGTCTCGGATTTTCGTAAGGACTTTCAGTTTTACAAGGTTCGGGAAATCAACAACTACCACCACGCACACGACGCTTACCTTAACGCAGTTGTCGGGACCGCGCTTATCAAAAAGTACCCCAAGCTTGAGTCCGAATTCGTTTACGGGGACTACAAGGTCTACGACGTCCGGAAGATGATCGCGAAATCGGAACAGGAGATTGGGAAGGCTACGGCTAAGTACTTTTTCTACTCGAACATCATGAACTTTTTTAAGACCGAGATCACCCTTGCTAACGGGGAAATCCGTAAGCGTCCCCTTATCGAGACCAACGGAGAGACCGGGGAGATCGTTTGGGACAAGGGACGTGACTTCGCAACCGTTCGTAAGGTTCTTTCCATGCCTCAGGTTAACATCGTTAAAAAGACCGAGGTTCAGACCGGGGGGTTTTCCAAAGAATCGATCCTCCCCAAGAGGAACTCCGATAAACTTATCGCGCGGAAGAAGGATTGGGACCCCAAGAAGTACGGGGGGTTCGACAGCCCCACGGTTGCGTACTCGGTTCTTGTTGTCGCGAAAGTTGAGAAGGGAAAGTCCAAAAAACTCAAGTCCGTCAAGGAACTCCTTGGAATCACCATCATGGAGAGGAGCTCCTTCGAGAAGAACCCTATCGATTTCCTCGAGGCGAAGGGGTACAAAGAGGTCAAGAAGGATCTCATCATCAAGCTTCCCAAGTACAGCCTTTTCGAACTCGAGAACGGGCGGAAGCGAATGTTGGCGAGCGCAGGGGAGCTCCAGAAGGGGAACGAATTGGCGTTGCCCTCCAAGTACGTTAACTTCCTCTACCTCGCGAGCCACTACGAGAAACTTAAAGGGAGCCCCGAGGACAACGAGCAGAAGCAGCTTTTCGTTGAACAGCACAAACACTACCTCGACGAAATCATCGAACAGATCAGCGAGTTCTCGAAACGGGTCATCCTCGCTGACGCAAACCTCGACAAGGTCCTTAGCGCTTACAACAAGCACAGGGATAAGCCCATTCGGGAGCAGGCTGAGAACATCATCCACCTCTTCACGCTTACGAACCTTGGGGCGCCTGCGGCGTTCAAGTACTTCGACACCACCATCGACCGGAAGCGTTACACGTCGACCAAGGAGGTCCTCGACGCAACCCTTATCCACCAGTCCATCACCGGGCTTTACGAGACCCGTATCGACCTTAGCCAGCTTGGGGGGGATTAA

**Table S5**. List of spacers used for targeting *Rhodobacter sphaeroides’* genomic DNA

| **Spacer name** | **Sequence** | **PAM** | **Targeted gene** |
| --- | --- | --- | --- |
| NT | AGGAGGGACGTTGCGACAAG | --- |  |
| sp1 | CACCTTCGCATAATATTGCA | CGG | *upp* |
| sp2 | ATCGATCTCCTGAAGGAGAA | GGG | *upp* |
| sp3 | CGGCGTCTGGATCGTCGTCG | TGG | *upp* |
| sp4 | CCGACCATGCTTGACCATTA | AGG | *Δupp* |
| sp5 | TAAAGCGGGGTTTCCCTTAA | TGG | *Δupp* |
| sp1 | GACCTACAAATGGTCCGTCG | CGG | *phaB* |
| sp2 | CGGGCAGGCGAACTATTCGG | CGG | *phaB* |
| sp3 | CAATCTCCTCCGGTTCGCCG | AGG | *phaB* |
| sp1 | CGGTCCGAAGCAGCTGCCGA | AGG | *phbB* |
| sp2 | TCGGCAGCTGCTTCGGACCG | CGG | *phbB* |

**Table S6**. Effect of the *phaB* (RSP_0747) and the *phbB* (RSP_3963) knockout, as well as of the combined knockout, on the C/N ratios the acids production and the PHB accumulation in *Rhodobacter sphaeroides* under nitrogen excess and limiting conditions on a defined medium.

|  | **Active biomass (g/L)** | **PHB (g/L)** |
| --- | --- | --- |
| **WT** | 0.81 ± 0.12 | 0.34 ± 0.018 |
| **Δ*phaB*** | 0.69 ± 0.11 | 0.002 ± 0.002 |
| **Δ*phbB*** | 0.79 ± 0.09 | 0.35 ± 0.0035 |
| **Δ*phaB* Δ*phbB*** | 0.78 ± 0.11 | 0.0001 ± 0.00 |
